# Position-Enhanced Multi-Head Self-Attention Based Bidirectional Gated Recurrent Unit for Aspect-Level Sentiment Classification

**DOI:** 10.3389/fpsyg.2021.799926

**Published:** 2022-01-25

**Authors:** Xianyong Li, Li Ding, Yajun Du, Yongquan Fan, Fashan Shen

**Affiliations:** ^1^School of Computer and Software Engineering, Xihua University, Chengdu, China; ^2^Sichuan Suitang Science and Technology Co., Ltd., Chengdu, China

**Keywords:** aspect-level sentiment classification, attention mechanism, position information, BiGRU, long short term memory networks, aspect terms

## Abstract

Aspect-level sentiment classification (ASC) is an interesting and challenging research task to identify the sentiment polarities of aspect words in sentences. Previous attention-based methods rarely consider the position information of aspect and contextual words. For an aspect word in a sentence, its adjacent words should be given more attention than the long distant words. Based on this consideration, this article designs a position influence vector to represent the position information between an aspect word and the context. By combining the position influence vector, multi-head self-attention mechanism and bidirectional gated recurrent unit (BiGRU), a position-enhanced multi-head self-attention network based BiGRU (PMHSAT-BiGRU) model is proposed. To verify the effectiveness of the proposed model, this article makes a large number of experiments on SemEval2014 restaurant, SemEval2014 laptop, SemEval2015 restaurant, and SemEval2016 restaurant data sets. The experiment results show that the performance of the proposed PMHSAT-BiGRU model is obviously better than the baselines. Specially, compared with the original LSTM model, the Accuracy values of the proposed PMHSAT-BiGRU model on the four data sets are improved by 5.72, 6.06, 4.52, and 3.15%, respectively.

## 1. Introduction

In natural language processing (NLP), the purpose of sentiment analysis (Pang and Lee, 2008) is to divide the texts into two or more sentiment categories (such as positive, neutral, and negative) based on the meaningful information from some texts. The aspect-level sentiment classification (ASC) is an important fine-grained sentiment classification. Its aim is to predict sentiment polarities of different aspect terms in a sentence (Thet et al., 2010). For example, in the sentence: “The environment of this restaurant is beautiful and the food is delicious, but the service is terrible,” the sentiment polarities of the aspect terms “environment,” “food,” and “service” are positive, positive, and negative, respectively. Since the traditional sentiment analysis only consider the polarities of sentiment for sentences (Mullen and Collier, 2004), the ASC is more complicated than traditional sentiment classification.

In machine learning models, a series of features, e.g., a set of words and sentiment dictionaries (Jiang et al., 2011; Zhang and Lan, 2015), were set up to train classifiers, such as SVM and KNN. Their classification effect heavily depended on the features' quality. Another more important models are deep learning models (Zhang et al., 2018). Because they did not deliberately design feature engineering, they can be effectively applied to automatically achieve the task of the ASC (Tang et al., 2016b). In recent, the recurrent neural network (RNN) (Socher et al., 2011; Nguyen and Shirai, 2015; Liu et al., 2016) and its variant models have been widely used in ASC tasks. These models can capture the relationships between sequences. Lai et al. (2015) used a two-way loop structure to obtain text information. Compared with traditional window-based neural networks, their method reduced more noise. Their method also retained the word order in a large range when it learned text expressions. For targeted sentiment classification, Gan et al. (2020) put forward a sparse attention mechanism based on a separable dilated convolution network. Their method is superior to the existing methods. Tang et al. (2016a) proposed a target-dependent long-term short-term memory network (TD-LSTM). This network is modeled by the contexts before and after the target word. By combining the information of the two LSTM hidden layer states, they further achieved the ASC tasks. Compared with the RNN model, the performances of these RNN variant models have small improvements on the ASC task.

For specific aspect terms in a sentence, the RNN model paid little attention to its contextual information. Based on visual attention (Mnih et al., 2014), the attention mechanism is extensively borrowed in neural networks (Luong et al., 2015; Yin et al., 2015; Liu and Lane, 2016). A lot of attention-based neural network models (Yin et al., 2015; Wang et al., 2016; Ma et al., 2017; Zeng et al., 2019) are proposed to solve ASC tasks. For a sentence, the attention mechanism makes the neural network model pay more attention to the sentiment descriptions of specific aspects, i.e., the sentiment polarities of aspect words, while ignoring other noise words that are not related to the aspect words. Xu et al. (2020) proposed a multi-attention network. They used the global and local attention modules to obtain the interactive information of different granularities between aspect words and contexts. Chen et al. (2017a) proposed a recurrent attention network model on memory for sentiment classification. Their model is established on cognition grounded data. The proposed cognition-based attention mechanism can be applied in sentence-level and document-level sentiment analysis. Based on the attention mechanism and LSTM networks, Ma et al. (2017) proposed an interactive attention network (IAN) model. Their model obtained good performance on SemEval 2014. When the aspect terms contain more than one word, their method may lead to the loss of useful information. The self-attention mechanism (Letarte et al., 2018) could make sentiment analysis models pay more attention to the useful information of aspect terms in the context and the internal structure of sentences. It improved the performance of neural network models. Xiao et al. (2020) used multi-head self-attention to get the semantic and interactive information in sentences. They further proposed a multi-head self-attention based gated graph convolutional network model. Their model can effectively achieve aspect-based sentiment classification. Leng et al. (2021) modified the transformer encoder to propose the enhanced multi-head self-attention. Through this attention, the inter-sentence information can be encoded. Combining with the enhanced multi-head self-attention and BiLSTM or BiGRU, they proposed a sentiment analysis model which performed better than some baselines in some evaluation indices. Therefore, the attention mechanism is becoming more and more important in the ASC task.

In addition, the position information between the aspect terms and their contexts has been confirmed that it was capable of improving the accuracy of the ASC (Chen et al., 2017a; Gu et al., 2018). For the RNN model (Liu and Lane, 2016; Liu et al., 2016), the calculation at the current moment depends on the result at the previous moment. This will result in a lack of contextual semantic information for aspect words. Zhou et al. (2019) used R-Transformer to get this semantic information. They further combined the self-attention mechanism and position relationship to propose the position and self-attention mechanism-based R-Transformer network (PSRTN) model for the ASC. Their experiment results are better than some baseline models. It is, thus, clear that the position information needs to consider in the context attention calculation.

Based on the above observations, this article proposes a position-enhanced multi-head self-attention based BiGRU (PMHSAT-BiGRU) model which integrates the position influence vector, multi-head self-attention mechanism, and bidirectional gated recurrent unit (BiGRU). This model considers three influence factors for the ASC task: the keywords in aspect terms, the position relationship between aspect terms and context, and semantic information of the context. In order to avoid noise words and make better use of the keywords in the aspect, it uses a self-attention mechanism to calculate the attention scores of the aspect words and each word in the sentence. To better obtain the semantic information of the context, it also uses multi-head attention to learn the relevant information from different representation subspaces. Finally, the PMHSAT-BiGRU model will be evaluated on the SemEval2014 restaurant, SemEval2014 laptop, SemEval2015 restaurant, and SemEval2016 restaurant dataset. Abundant experiments will verify its effectiveness on the ASC task.

In general, the main contributions of this article are as follows:

(1) Based on the position information between the aspect terms and context, a positional information vector is designed. It uses the relative position method to participate in the calculation of the attention weight.
(2) To get a contextual representation of the specific aspect terms, a self-attention mechanism is used to calculate the words' weights in aspect terms. The multi-head attention mechanism is employed to represent the semantic information of the context in different representation subspaces.
(3) A PMHSAT-BiGRU model is proposed. Considering that three main factors, including the keywords in aspect terms, the position relationship between aspect terms and context, and the semantic information of the context for a sentence, affect the ASC, the PMHSAT-BiGRU model integrates the position influence vector, multi-head self-attention mechanism, and BiGRU.
(4) Extensive experiments on four datasets including SemEval2014 restaurant, SemEval2014 laptop, SemEval2015 restaurant, and SemEval2016 restaurant data sets are conducted. The performance of the PMHSAT-BiGRU model is evaluated by using the Accuracy (Acc) and Macro-Average F1 (Macro-F1).

The rest of this article is organized as follows. Section 2 introduces the related work of the ASC. Section 3 elaborates the proposed PMHSAT-BiGRU model. In section 4, we carry out a large number of experiments to prove the validity of the proposed model. Finally, we make the summary and forecast to the full text in section 5.

## 2. Related Work

The ASC focuses on the sentiment polarities of aspect terms in a sentence. Since neural network models (Santos and Gattit, 2014; Zhang et al., 2018; Chen and Huang, 2019) are superior to the machine learning methods (Mullen and Collier, 2004; Jiang et al., 2011; Zhang and Lan, 2015) in sentiment classification, many new research results are based on neural networks. On the basis of the RNN (Mikolov et al., 2010; Akhtar et al., 2020), Hochreiter et al. explored the long short-term memory network (LSTM) (Hochreiter and Schmidhuber, 1997) and the gated recurrent unit (GRU) (Dey and Salemt, 2017). These models could solve the gradient descent and explosion problems. Tang et al. (2016a) integrated the information of the target words and context words to establish the sentence semantically. They presented two improved LSTM models, i.e., the target-dependent LSTM and target-connection LSTM. These models are significantly superior to the original LSTM model. Jiang et al. (2011) took the content, sentiment lexicon and context into consideration to improve the target-dependent sentiment classification for Twitter. Tan et al. (2020) proposed an aligning aspect embedding method to train aspect embeddings for the ASC. The embeddings are applied to the gated convolutional neural networks (CNNs) and attention-based LSTM. Their experiment results showed that the model with the aspect embedding obtained better performance than other baseline models. Xue and Li (2018) proposed Gated Tanh-Rectified Linear Unit (ReLU) Units. They further built a new CNN model with this mechanism to predict the sentiment polarities of aspect terms. The training time of the model was faster than other baseline models.

The attention mechanism and position information are also considered in different neural network models for the ASC. Wang et al. (2016) designed a novel attention mechanism to capture the vital part of sentences with different aspect terms. Based on this mechanism, they presented an ATAE-LSTM model to effectively achieve the binary and 3-class prediction problems in the ASC. Considering the explicit memory, position, and context attentions, Tang et al. (2016b) designed deep memory networks. To a certain extent, their models achieved good performance on the ASC tasks. Liu et al. (2015); Chen et al. (2017b) introduced position information into attention mechanism to handle tasks of question answering and machine translation. The performance of the two tasks was obviously improved.

Although these models have provided a good performance on the ASC tasks, the neural network models with position relationships and multi-head self-attention mechanism have yet to be studied for the ASC.

## 3. PMHSAT-BiGRU for the ASC

**Figure 1:**
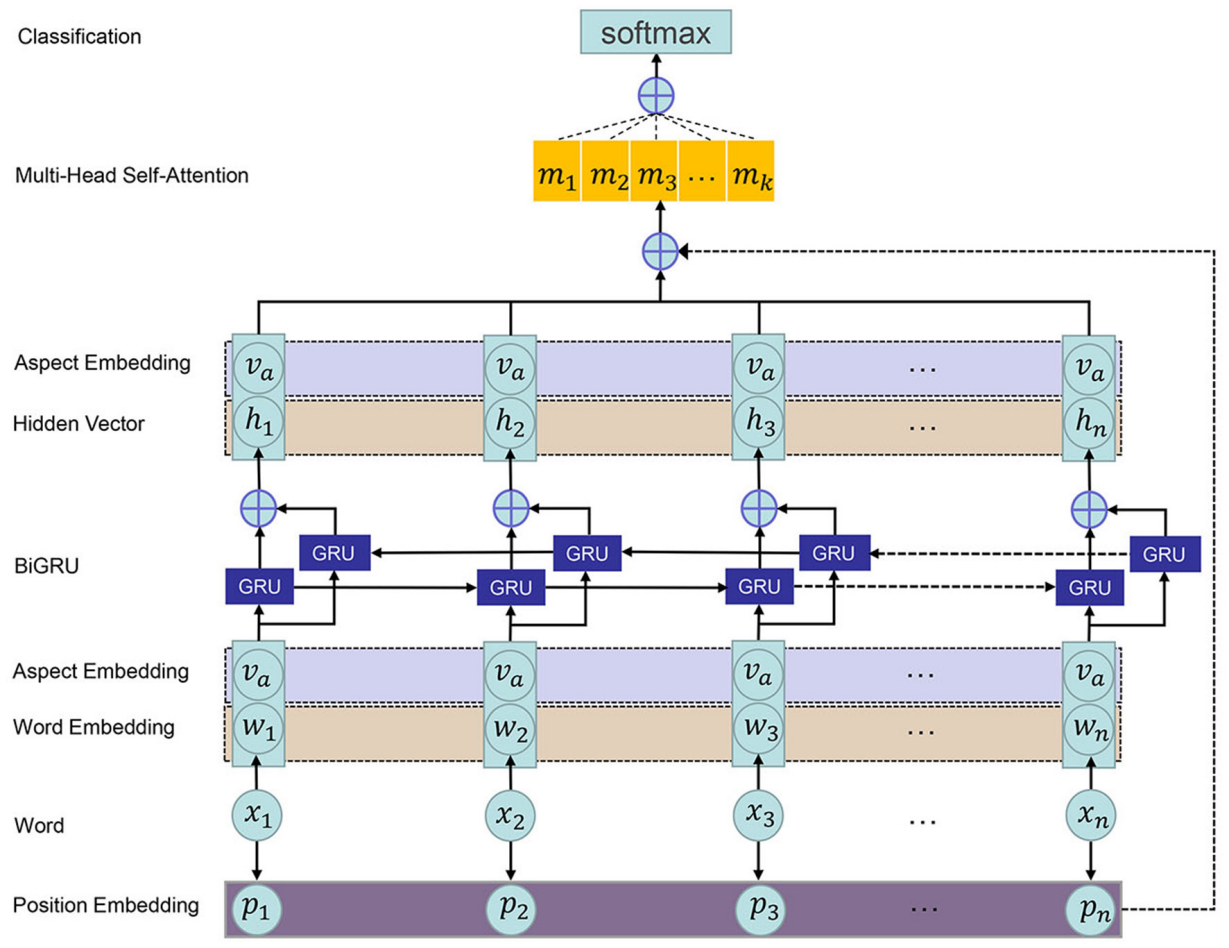
The architecture of the PMHSAT-BiGRU for ASC.

In this section, we will minutely describe the PMHSAT-BiGRU model (refer to Figure 1), including the task definition, position modeling, word representation, BiGRU, attention mechanism, sentiment classification, and model training.

### 3.1. Task Definition

For a sentence with N-words and M aspect terms, let 〈*aspect term i, sentence*〉 be the aspect-sentence pair for the aspect term *i, i* = 1, 2, ⋯, *M*. Then, using 〈*aspect term i, sentence*〉 as an input of the ASC, the sentiment category 〈*positive, neural, negative*〉 will be predicted for the aspect term *i* in the sentence. For example, the sentence “Great food but the service was dreadful!” involves two aspect terms, namely [food] and [service]. The sentence will generate two aspect-sentence pairs including 〈*food, sentence*〉 and 〈*service, sentence*〉 as the inputs of the ASC, then expectation outputs of the aspect terms [food] and [service] are positive and negative, respectively.

### 3.2. Position Modeling

In the ASC task, the sentiment polarity of a particular aspect will be severely affected by adjacent context words in a sentence. Inspired by Shaw et al. (2018), we employ relative position to model the position information of the aspect words in the corresponding sentence. For a sentence with aspect terms, the position indices of the words contained in an aspect term are marked as “0,” and the position indices of other words will be expressed as the relative distances from the current aspect term. Therefore, the position index of a word for the sentence is the following:


(1) $$p_{i}=\left\{ \begin{array}{l} \begin{array}{cc} |i-a_{start}|, & i<a_{start}, \end{array}\\ \begin{array}{cc} 0, & a_{start}\leq i\leq a_{end}, \end{array}\\ \begin{array}{cc} |i-a_{end}|, & i<a_{end}, \end{array} \end{array}\right.$$

where *a*_*start*_ and *a*_*end*_ respectively represent the start and end indices of the aspect term; and *p*_*i*_ represents the relative distance from the ith word to the aspect term in the sentence. According to these indices from the first word to the last word in the sentence, a position index sequence with the sentence length of *N* is *p* = [*p*_1_, *p*_2_, ⋯, *p*_*N*_] for an aspect term. For example, in the sentence “The seafood menu is interesting and quite reasonably priced.”, there are two aspect terms “seafood menu” and “priced.” Then the position index sequences of “seafood menu” and “priced” are expressed as *p* = [1,0,0,1,2,3,4,5,6] and *p* = [8,7,6,5,4,3,2,1,0], respectively. By looking up the position embedding matrix $P\in {\mathbb{R}}^{d_{p}\times N}$, the corresponding position embeddings are obtained, where *d*_*p*_ is the dimension of the position embedding, and *N* is the length of the sentence. Then, the position embeddings are randomly initialized and updated during the training process. After transforming the position indices into position embeddings, the embeddings can model the different weights of words with different distances. In the example above, the sentiment word “interesting” is more important than the words “quite reasonably” for the aspect term “seafood menu.” It implies that when an aspect term needs to predict its sentiment polarity, the words with relatively small distances and sentiment polarities are more important than other words.

### 3.3. Word Representation

By word embedding technology, each word is embedded into a unique word vector with the information of the word itself in the vector space. To obtain the word embedding, we will apply Glove (Pennington et al., 2014) pre-trained at Stanford University. In the following, all word embeddings are denoted by $E\in {\mathbb{R}}^{d_{w}\times |V|}$, where *d*_*w*_ represents the dimension of the word embeddings and |*V*| represents the size of the vocabulary. All aspect embeddings are expressed as $A\in {\mathbb{R}}^{d_{a}\times |L|}$, where *d*_*a*_ is the dimension of aspect embeddings, and |*L*| is the size of aspect terms. For a sentence with *N*-words [*w*_1_, *w*_2_, ⋯, *w*_*N*_], if it contains an aspect term [*a*_1_, *a*_2_, …, *a*_*M*_] with *M* words, then the sentence embedding and the aspect embedding will be obtained by finding the embedding matrix *E* and *A*, respectively.

### 3.4. Bidirectional Gated Recurrent Unit

Recurrent neural network has been successfully applied in the field of the NLP. However, the standard RNN often faces the problem of gradient disappearance or gradient explosion. As a special RNN, LSTM adjusts the cell state through three gated mechanisms at each time step, better solving the problem of the long dependence. Compared with the one-way LSTM, BiLSTM can learn more contextual information. It establishes the context dependence in the forward and reverse directions. Concretely, the forward LSTM processes sentences from the left to the right, and the reverse LSTM processes sentences from the right to the left. From this, it gets two hidden representations, and then connects the forward hidden state and backward hidden state of each word as the final representation.

In contrast with the LSTM, the GRU, which uses two gated mechanisms to adjust cell state and has fewer parameters and lower computational complexity has relatively better performance than LSTM in the NLP. Specifically, at time *t*, we obtain the embedding vector $w_{t}\in {\mathbb{R}}^{d_{w}}$ of the current input word from *E* and the aspect embedding vector $v_{a}\in {\mathbb{R}}^{d_{a}}$ from *A*, then the current hidden layer vector *h*_*t*_ in GRU is updated by the following:


(2) $$\begin{array}{l} z_{t}=\sigma (W_{z}h_{t-1}+U_{z}[w_{t},v_{a}]+b_{z}),\\ r_{t}=\sigma (W_{r}h_{t-1}+U_{r}[w_{t},v_{a}]+b_{r}),\\ \tilde{h_{t}}=\tanh(W_{h}(h_{t-1}⊙r_{t})+U_{h}[w_{t},v_{a}]+b),\\ h_{t}=h_{t-1}⊙(1-z_{t})+z_{t}⊙\tilde{h_{t}}, \end{array}$$

where *z* and *r* are the update gate and reset gate, respectively; the sigmoid function σ(·) is used to control the retention of useful information and the discarding of the useless information; $W_{z},W_{r},W_{h}\in {\mathbb{R}}^{d_{h}\times d_{h}},U_{z},U_{r},U_{h}\in {\mathbb{R}}^{d_{h}\times \left(d_{w}+d_{a}\right)},b_{z},b_{r},b\in {\mathbb{R}}^{d_{h}}$ represent the weight matrices and biases learned in the GRU training process; ⊙ denotes an element multiplication; and [*w*_*t*_, *v*_*a*_] stands for the splicing vector of the word embedding *w*_*t*_ and the aspect embedding *v*_*a*_. Then, the hidden vector [*h*_1_, *h*_2_, …, *h*_*N*_] of the sentence with the length *N* is regarded as the final context word representation.

In the following, we will adopt the BiGRU to obtain the contextual representation of a sentence. Compared with the one-way GRU, BiGRU includes the forward hidden state $\vec{h_{i}^{t}}\in {\mathbb{R}}^{d_{h}}$ and the backward hidden state $\overset{⃖}{h_{i}^{t}}\in {\mathbb{R}}^{d_{h}}$ at time *t*, where *d*_*h*_ represents the number of hidden layer units. Then, the forward hidden state $\vec{h_{i}^{t}}$ and the backward hidden state $\overset{⃖}{h_{i}^{t}}$ are connected as the final context hidden representation $h_{i}^{t}=\left[\vec{h_{i}^{t}};\overset{⃖}{h_{i}^{t}}\right]\in {\mathbb{R}}^{2d_{h}}$.

### 3.5. Attention Mechanism

The attention mechanism can help the model focus on the important parts of a sentence in the ASC tasks. In particular, the multi-head attention mechanism allows the model to learn some relevant information in different representation subspaces. Furthermore, the self-attention mechanism can learn the word dependency relationships within the sentence and then capture the internal structure of the sentence. This mechanism can process in parallel, reducing the complexity of calculations. In view of these advantages, the overall semantics of a sentence can be represented by the multi-head self-attention mechanism (Zhou et al., 2019). Based on the last hidden layer state $h_{i}^{t}$ output by BiGRU, the current context representation can be represented as $h_{1}^{t},h_{2}^{t},\cdots \;,h_{N}^{t}$. Then, feeding them into the multi-head self-attention, a new representation *s*_*t*_ for the sentence can be obtained by the following:


(3) $$\begin{array}{l} s_{t}=MultiHeadAttention(h_{1}^{t},h_{2}^{t},\cdots \;,h_{N}^{t})\\ \;=Concat(head_{1}(h_{N}^{t}),head_{2}(h_{N}^{t}),\cdots \;,head_{k}(h_{N}^{t}))W^{o},\; \end{array}$$

where ${head}_{i}\left(h_{N}^{t}\right)$ denotes the value of the *i*-th attention head; and *W*^*o*^ stands for the linearization mapping matrix. For ${head}_{i}\left(h_{N}^{t}\right)\left(i=1,2,\cdots \;,N\right)$, it is calculated by the following formulas:


(4) $$\begin{array}{c} \alpha_{1},\alpha_{2},\cdots \;,\alpha_{N}=softmax\left(\frac{QK^{T}}{\sqrt{d_{k}}}\right)V,\\ {head}_{i}\left(h_{N}^{t}\right)=\overset{N}{\underset{j=1}{\sum }}\alpha_{j}v_{j}, \end{array}$$

where *Q*, *K*, and *V* represent the query, key, and value matrices, respectively. In these matrices, their vectors *q, k*_*i*_ and *v*_*i*_ are calculated as follows:


(5) $$\begin{array}{l} q,k_{i},v_{i}=W^{q}h_{N}^{t},W^{k}h_{i}^{t},W^{v}h_{i}^{t}, \end{array}$$

where *W*^*q*^, *W*^*k*^, and *W*^*v*^ are the weight matrices whose values are different in different attention heads.

### 3.6. Sentiment Classification

For the multi-head self-attention representation *s*_*t*_, we map it to the target space with *C* sentiment polarities by a non-linear layer:


(6) $$\begin{array}{l} x=\tanh\left(W_{r}s_{t}+b_{r}\right), \end{array}$$

where *x* = (*x*_1_, *x*_2_, ⋯, *x*_*C*_), *W*_*r*_ and *b*_*r*_ are the weight matrix and bias within the non-linear layer, respectively. Then, *x* is transformed into the conditional probability distribution through a Softmax layer. Therefore, the final distributions of the *C* sentiment polarities are obtained by the following:


(7) $$y_{c}=\frac{exp(x_{c})}{\sum_{c=1}^{C}exp(x_{c})}.$$

From this result, the sentiment polarity corresponding to the maximum probability, i.e., $max_{c=1}^{C}\left\{y_{c}\right\}$, is chosen as the final sentiment classification.

### 3.7. Model Training

In the PMHSAT-BiGRU model, the cross entropy and *L*_2_ regularization


(8) $$\begin{array}{l} L=-\underset{d\in D}{\sum }\overset{C}{\underset{c=1}{\sum }}y_{c}\left(d\right)log\left(g_{c}\left(d\right)\right)+\frac{1}{2}\lambda {∥\theta ∥}^{2}, \end{array}$$

will be regarded as the loss function, where *D* denotes the data set which consists of different sample *d*; $y_{c}\left(d\right)\in {\mathbb{R}}^{C}$ represents the real sentiment polarity distribution of sample *d*; $g_{c}\left(d\right)\in {\mathbb{R}}^{C}$ stands for the sentiment polarity vector of sample *d*; λ is the *L*_2_ regularization coefficient; and θ includes all model parameters. For the sake of optimizing all model parameters, the loss function should be minimized as much as possible. By the back-propagation method, the parameters θ is updated by the following:


(9) $$\begin{array}{l} \theta =\theta -\lambda_{l}\frac{\partial L\left(\theta \right)}{\partial \theta }, \end{array}$$

where λ_*l*_ is the learning rate. In order to prevent overfitting during training process, the dropout strategy is adopted as the method of discarding some learned features.

## 4. Experiments

In this section, we will make some experiments under the proposed PMHSAT-BiGRU model and several baseline models on several large data sets. By comparing the results of these experiments, the effectiveness of the proposed PMHSAT-BiGRU model will be verified. Then several ablation experiments are set to affirm the effectiveness of the modules in the proposed model. Finally, we visualize the dataset in the experiment based on the proposed PMHSAT-BiGRU model.

### 4.1. Experimental Setting

#### 4.1.1. Dataset

The ASC benchmark data sets, officially published by SemEval including SemEval 2014 Task4^1^, SemEval 2015 Task12^2^, and SemEval 2016 Task5^3^, will be adopted. In these datasets, the SemEval 2014 contains Restaurant14 (R14) and Laptop14 (L14) datasets; the SemEval 2015 uses Restaurant15 (R15) dataset; and the SemEval 2016 uses the Restaurant16 (R16) dataset. More specifically, each dataset contains a training set and a test set. In each dataset, every data is a single sentence, including the review text, aspect terms, sentiment labels corresponding to the aspect terms, and the starting position of the aspect terms. There are four aspect-level sentiment polarities, i.e., positive, negative, neutral, and conflict in these data sets. To facilitate subsequent experiments, we only use the positive, negative, and neutral aspect-level sentiment polarities and remove the conflict aspect-level sentiment polarity from these data sets, i.e, the number of the sentiment polarity categories *C* = 3. For all adopted datasets, their details of the training sets and test sets are shown in Table 1. In addition, we count the number of words in the aspect terms in Table 2. It easily finds that more than one-fourth of the datasets have the aspect terms with multiple words.

**Table 1:** Samples of semeval 2014–2016 datasets.

**Datasets**		**Positive**	**Negative**	**Neutral**
R14	Train	2,164	807	637
	Test	728	196	196
L14	Train	994	870	464
	Test	341	128	169
R15	Train	1,178	382	50
	Test	439	328	35
R16	Train	1,620	709	88
	Test	597	190	38

**Table 2:** The numbers of terms in statistical datasets.

**Datasets**		**Len = 1**	**Len = 2**	**Len ≥3**
R14	Train	2,720 (75.38%)	604 (16.74%)	284 (7.87%)
	Test	801 (71.52%)	215 (19.20%)	104(9.29%)
L14	Train	1,473 (63.27%)	649 (27.88%)	206 (8.85%)
	Test	351 (52.78%)	209 (31.43%)	78 (11.73%)
R15	Train	1,272 (79.00%)	216 (13.41%)	122 (9.59%)
	Test	638(79.55%)	94(11.72%)	70(8.73%)
R16	Train	1,941 (80.31%)	301 (12.45%)	175 (7.24%)
	Test	668 (80.97%)	101 (12.24%)	56 (6.79%)

#### 4.1.2. Parameters Setting

In our experiments, the Glove^4^ (Pennington et al., 2014) is used to initialize the aspect and contextual word embedding. It sets the embedding dimension of each word to 300. The weight matrices are initialized from the uniform distribution *U*(−μ, μ), where μ = 0.01 and all offsets are set to 0. The aspect embedded dimension is also set to 300; the BiGRU hidden unit is set to 200; and the position embedded dimension is set to 100. The maximum length of a sentence is 80; the batch size is 4; and the number of multi-head self-attention heads is 8. In our PMHSAT-BiGRU model, the dropout rate is set as 0.5; the *L*_2_ regularization coefficient is set as 1e-5; the Adam optimizer is used to optimize the training parameters; and the learning rate is set as 1e-4. To implement our PMHSAT-BiGRU model, we employ Pytorch^5^ in the experiments.

#### 4.1.3. Evaluation

In the experiments, we used two common evaluation indexes, i.e., Acc and Macro-F1 in classification tasks. In detail, the Acc represents the proportion of correctly classified samples to the total sample number, and its calculation is as follows:


(10) $$\begin{array}{l} Accuracy=\frac{tp+tn}{tp+tn+fp+fn}, \end{array}$$

where *tp* denotes the number of the samples whose true labels and sentiment labels predicted by the model are both positive categories; and *tn* represents the number of the samples whose true labels are positive categories and sentiment labels predicted by the model are negative categories. Correspondingly, *fp* represents the number of the samples whose true labels are negative categories and sentiment labels predicted by the model are positive categories; and *fn* represents the number of the samples whose true labels and sentiment labels predicted by the model are both negative categories.

Next, the Recall, Precision, and F1-score (RPF value) are calculated by the following.


(11) $$\begin{array}{l} Recall=\frac{tp}{tp+fn},\\ Precision=\frac{tp}{tp+fp},\\ F1-score=\frac{2\times Precision\times Recall}{Precision+Recall}. \end{array}$$

In the experiments, we will calculate the RPF values for the positive, negative, and neutral categories. Then, we obtain the Macro-F1 values by averaging the F1-score values of the three categories.

### 4.2. Baselines

In order to verify the effectiveness of the PMHSAT-BiGRU model, the experiment results will compare with the following baseline models:

**Context word vectors average (ContextAvg)**: It averages the word embedding and aspect vectors, and then input the result into the softmax classifier, which was cited as the baseline model in Tang et al. (2016b).

**Long short-term memory (LSTM) (Hochreiter and Schmidhuber**, **1997****)**: For a sentence, the one-way LSTM network is used to model the sentence; the last hidden layer vector is regarded as the final representation of the sentence, and then sent to the Softmax classifier for the final classification.

**Target-dependent long-term short-term memory (TD-LSTM) (Tang et al.**, **2016a****)**: For a sentence with target words, the sentence is divided into two different parts based on a target word of the sentence, and then respectively uses two LSTMs to model the context on the left side of the target word and the right side of the target word. Finally, it connects the related representations of the two parts as the classifier input to predict the sentiment polarity of the target word.

**Target-connection long-term short-term memory (TC-LSTM) (Tang et al.**, **2016a****)**: This model is similar to TD-LSTM. However, the difference is that TC-LSTM has added the aspect word information at the input; and the word vector and the aspect vector are connected, obviously integrating the correlation information between the aspect word and the context word.

**Attention-based long short-term memory (AE-LSTM) (Wang et al.**, **2016****)**: Based on the standard LSTM, the aspect embeddings are designed to represent the aspect information; and the aspect embeddings are regarded as a part of the training parameters.

**Attention-based long short-term memory with aspect embedding (ATAE-LSTM)**: On the basis of AE-LSTM, the aspect is embedded in each word embedding and hidden vector; and the attention mechanism is used to further strengthen the effect of the aspect embedding. This model was cited as the baseline model in Zhou et al. (2020).

**Memory Network (MemNet)**: Using the deep memory network instead of the RNN-based method for sentence modeling, it repeatedly employs the attention mechanism to capture the connections between the context words and aspect words. This model was cited as the baseline model in Zhou et al. (2020).

**Interactive attention network (IAN) (Ma et al.**, **2017****)**: Two LSTMs are respectively used to model the aspect terms and context words. Through the interactive attentions from the sentences to their corresponding aspects and from the aspects to the sentences, the sentence representations and aspect representations are generated. Then, the two representations are connected to input the Softmax classifier for the classification.

**Gated convolutional network with aspect embedding (GCAE) (Xue and Li**, **2018****)**: Many pairs of convolution kernels are used to extract local N-gram features, where each pair of convolution kernels contains one aspect-independent convolution kernel and one aspect-dependent convolution kernel. Then, the model respectively adopts tanh and ReLU gated units to output the sentiment features of a given aspect.

**Attention-based long short-term memory with position context (PosATT-LSTM) (Zeng et al.**, **2019****)**: On the basis of the one-way LSTM, the position relationships between the aspect words and the context are considered. The relationships are applied to the calculations of the attention weights.

### 4.3. Compared Methods

Based on the proposed PMHSAT-BiGRU model and baseline models, some experiments on R14, L14, R15, and R16 are made. These models' Acc and Macro-F1 values are shown in Table 3.

**Table 3:** The results of the PMHSAT-BiGRU model and baseline models in Semeval2014–2016.

	**R14**		**L14**		**R15**		**R16**	
	**Acc**	**Macro-F1**	**Acc**	**Macro-F1**	**Acc**	**Macro-F1**	**Acc**	**Macro-F1**
ContextAvg	71.53	58.02	61.59	53.92	73.79	47.43	79.87	55.68
LSTM	74.80	59.08	67.08	60.53	75.15	51.27	80.09	55.09
TD-LSTM	77.30	63.33	68.10	62.02	77.28	59.04	82.56	56.15
TC-LSTM	76.60	61.52	68.30	62.26	76.44	57.65	81.90	55.01
AE-LSTM	76.12	61.08	68.83	62.08	75.94	50.11	82.26	56.96
ATAE-LSTM	78.12	68.40	69.44	62.45	78.34	58.47	83.24	61.99
MemNet	78.16	65.83	70.33	64.09	77.89	59.52	83.04	57.91
IAN	78.60	66.31	72.10	65.92	78.62	55.34	82.19	56.30
GCAE	77.41	65.06	69.12	62.17	78.25	54.31	82.35	56.49
PosATT-LSTM	79.40	-	72.80	-	-	-	-	-
PMHSAT-BiGRU	**80.27**	**69.25**	**73.14**	**68.27**	**79.67**	**61.89**	**83.24**	**62.39**

*The bold value indicates that the effect of this method is the best compared with other baseline models*.

From Table 3, compared with other models, the performance of the ContextAvg model is the worst because it is directly classified by average word embedding and aspect embedding. Among the sequential models, the performance of the LSTM model is the worst because the model does not consider the attention mechanism and aspect word information but equally treats the aspect words and other words in the model. Compared with the LSTM model, the aspects are embedded into the LSTM model for training in the AE-LSTM model. So, the Accuracy values under the AE-LSTM model are respectively 1.32, 1.75, 0.79, and 2.17% better than the values under the LSTM model on R14, L14, R15, and R16. Compared with the TD-LSTM model, although the TC-LSTM model considers the aspect word information at the input end, its performance is worse than the TD-LSTM model.

Because the attention mechanism is used to model the relationships between the aspect words and context under the Memnet model, the performance of the Memnet model is better than the AE-LSTM model. Compared to the Memnet and ATAE-LSTM model, the Accuracy and Macro-F1 values on the Memnet model are slightly higher than the values on the ATAE-LSTM model under partial datasets. The performance of the IAN model is better than the ATAE-LSTM model because, in the IAN model, two LSTMs are respectively adopted to model the aspect terms and context, and an interactive attention mechanism is used to obtain context related to aspect terms. However, the importance of the position relationships between aspect words and context is not considered in the IAN model. Therefore, the performance of the IAN model is worse than the PosATT-LSTM model. Because the GCAE model uses the CNN and gated mechanism to realize parallel computation, making the model insensitive to position information, its performance on R16 has little promotion compared with the IAN model.

For the PMHSAT-BiGRU model, the aspect embedding information and the importance of the aspect words and context position information are applied in the calculations of the attention weights. Meanwhile, the multi-head attention mechanism is used to learn the dependent information in different contexts. The self-attention mechanism is also employed to capture the important words in the aspect terms. For the PosATT-LSTM model, only the semantic representation in a single context is captured; and each word in aspect terms is equally treated. Therefore, the performance of the PMHSAT-BiGRU model is obviously better than the PosATT-LSTM.

Overall, the performance of the PMHSAT-BiGRU model is superior to above baseline models. In particular, compared with the original LSTM model, the Accuracy values of the PMHSAT-BiGRU model on R14, L14, R15, and R16 are improved by 5.72, 6.06, 4.52, and 3.15%, respectively.

### 4.4. Model Analysis

In this section, a series of models will be designed to verify the effectiveness of the PMHSAT-BiGRU model. First of all, in order to verify the validity of the position information, the position information is removed from the PMHSAT-BiGRU model, denoted by the MHSAT-BiGRU model. In the MHSAT-BiGRU model, the representations of aspect words and sentences, and the multi-head self-attention mechanism is adopted to model the relationships between aspect words and sentences. Second, in order to verify the effectiveness of the multi-head self-attention mechanism for the PMHSAT-BiGRU model, the multi-head self-attention mechanism is replaced with a normal attention mechanism and the other parts are kept unchanged in PMHSAT-BiGRU, denoted by the PAT-BiGRU model. The structure of the PAT-BiGRU model is almost similar to the ATAE-LSTM model, except that the PAT-BiGRU model considers the position relationship between aspect words and context and uses the BiGRU structure instead of LSTM. Finally, we also use the BiGRU model to verify the effectiveness of our multi-head self-attention mechanism. The experimental results of these models are shown in Table 4.

**Table 4:** Analysis of position-enhanced multi-head self-attention based BiGRU model (PMHSAT-BiGRU) model.

	**R14**	**L14**	**R15**	**R16**
BiGRU	77.14	69.44	76.92	81.55
MHSAT-BiGRU	78.31	70.06	77.28	81.96
PAT-BiGRU	79.38	71.32	77.75	82.75
PMHSAT-BiGRU	**80.27**	**73.14**	**79.67**	**83.24**

*The bold value indicates that the effect of this method is the best compared with other baseline models*.

From Table 4, the performance of the BiGRU model is the worst in all models. The reason is that this model equally treats every word in sentences. In contrast, the multi-head self-attention mechanism can learn contextual information related to terms from different contexts. So the MHSAT-BiGRU model gets better grades than the BiGRU model. Because the PAT-BiGRU model uses position embedding and aspect embedding to calculate the weight of attention, while the MHSAT-BiGRU model only adopts the aspect embedding, the PAT-BiGRU model performs better than the MHSAT-BiGRU model. Compared with the PMHSAT-BiGRU model, since the PAT-BiGRU model ignores the aspect words with different meanings in different contexts and the role of important words in aspect terms, the performance of the PAT-BiGRU model is lower than the PMHSAT-BiGRU model.

On the basis of the above analysis, the PMHSAT-BiGRU model performs the best in all models. The reason is that the model not only fully considers the position information of aspect terms in the corresponding sentences but also regards the relationships between aspect terms and sentences from multiple levels. Besides, the model pays more attention to the important words in aspect terms, which is mainly realized by the multi-head self-attention mechanism.

**Figure 2:**
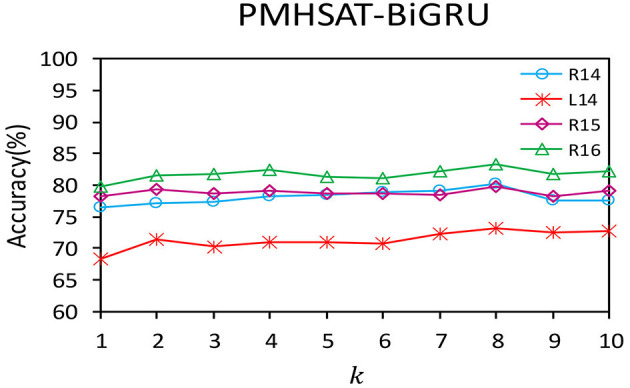
The influence of the number of the heads k of the multi-head attention mechanism on the accuracy of the model.

The multi-head self-attention mechanism is employed to learn the semantic information in different representation subspaces for the PMHSAT-BiGRU model, where the number of the subspaces is controlled by the number of the heads *k* in the multi-head attention mechanism. In the following, the influence of the parameter *k* on the Accuracy of the PMHSAT-BiGRU model is shown in Figure 2.

It can be observed that when *k* increases, the changing trends of the Accuracy values for the PMHSAT-BiGRU model on the four data sets are similar. Specifically, when *k* = 1, the multi-head self-attention mechanism is equivalent to an ordinary single-head self-attention mechanism. As the values of *k* increase, the performance of the model almost increases from 1 to 8, and then the performance of the model declines with the rise of *k*. The main reason is that when the value of *k* is more than 8, some heads will learn same attention weights, which bring noise for the sentiment classification of aspect terms. Evidently, when *k* = 8, the performance of the model on the four data sets is the best.

### 4.5. Case Study

**Figure 3:**
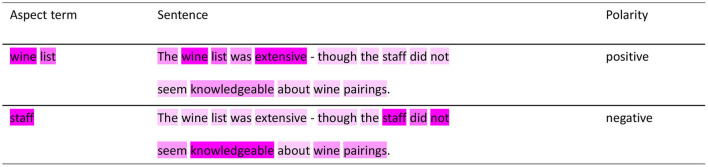
Visualize the weight of attention for aspect terms and sentences by PMHSAT-BiGRU.

In order to intuitively show the validity of the model, we will take a sentence with aspect terms as an example for predicting the aspect terms of sentences by the PMHSAT-BiGRU model. For example, the sentiment polarities of the sentence “The wine list was extensive-though the staff did not seem knowledgeable about wine pairings.” will be predicted by the model. For the sentence, the attention weights of the aspect terms and the sentence are visualized in Figure 3, the darker the color of words is, the more the words are important for predicting the sentiment polarities of aspect terms. It easily finds that the model focuses on the words adjacent to the aspect terms. When the model predicts the sentiment polarity of the aspect term “wine list,” the word “extensive” is closer to the position of the words “wine list,” so the model pays more attention to “extensive” which plays an important role in calculating the sentiment polarity of the aspect term “wine list;” whereas, the words “not” and “knowledgeable” are farther away from the aspect term, and then they receive less attention. In the aspect term “wine list,” the word “wine” gets more attention which is mainly realized by the self-attention mechanism. So the model can correctly predict the sentiment polarity of the aspect term “wine list” as positive. Similarly, when the model predicts the sentiment polarity of the aspect term “staff,” the words: “knowledgeable” and “not” get more attention than other words in the model. Since the positive polarity of the word “knowledgeable” for the “staff” is eventually reversed by the word “not,” the model can correctly predict the sentiment polarity of “staff” as negative. Therefore, the PHMSAT-BiGRU model accurately predicts the sentiment polarities of all aspect terms of the sentence. From this, even if a given sentence contains multiple aspect terms, the PMHSAT-BiGRU model can find the relevant sentiment descriptors of the given aspect terms, exactly predicting the sentiment polarities of its aspect terms.

## 5. Conclusion and Future Study

In this article, a PMHSAT-BiGRU based on the position influence vector, multi-head self-attention mechanism, and BiGRU is proposed for the ASC. The PMHSAT-BiGRU model considers the aspect terms contained in multi-words and the importance of each context word. The model also integrates the aspect word and its relative position information of the context into the semantic model. First, this model establishes position vectors based on the position information between the aspect words and its context. Then the position vectors and aspect embeddings are added to the hidden representations of BiGRU. Finally, the keywords in aspect terms and the sentiment features related to the aspect terms are captured by the multi-head self-attention mechanism. The experimental results on the SemEval 2014, 2015, and 2016 datasets show that the PMHSAT-BiGRU model can learn effective features and obtain better performance than the baseline model on the ASC tasks. In future study, the individual models and different fused approaches of the three important factors will be further improved.

## Data Availability Statement

The original contributions presented in the study are included in the article/supplementary material, further inquiries can be directed to the corresponding author/s.

## Author Contributions

XL and YD contributed to conception and design of the study. LD and XL made all experiments and wrote sections of the manuscript. YF and FS organized the database. All authors contributed to manuscript revision, read, and approved the submitted version.

## Funding

This study was partially supported by the National Natural Science Foundation (Nos. 61802316, 61872298 and 61902324), Chunhui Plan Cooperation and Research Project, Ministry of Education of China (Nos. Z2015109 and Z2015100), Young Scholars Reserve Talents program of Xihua University, Scientific Research Fund of Sichuan Provincial Education Department (No. 15ZA0130), Science and Technology Department of Sichuan Province (Nos. 22ZDYF3157 and 2021YFQ0008), Key Scientific Research Fund of Xihua University (No. z1422615), Xihua University Innovation Fund (Nos. YCJJ2021031, YCJJ2021025, and YCJJ2021124), and Opening Project of Intelligent Policing Key Laboratory of Sichuan Province (No. ZNJW2022ZZZD003).

## Conflict of Interest

FS was employed by company Sichuan Suitang Science and Technology Co., Ltd. The remaining authors declare that the research was conducted in the absence of any commercial or financial relationships that could be construed as a potential conflict of interest.

## Publisher's Note

All claims expressed in this article are solely those of the authors and do not necessarily represent those of their affiliated organizations, or those of the publisher, the editors and the reviewers. Any product that may be evaluated in this article, or claim that may be made by its manufacturer, is not guaranteed or endorsed by the publisher.

## Footnotes

^1^ https://alt.qcri.org/semeval2014/task4/

^2^ https://alt.qcri.org/semeval2015/task12/

^3^ https://alt.qcri.org/semeval2016/task5/

^4^ http://nlp.stanford.edu/projects/glove/

^5^ https://pytorch.org/
